# Simulation-based assessment of the performance of hierarchical abundance estimators for camera trap surveys of unmarked species

**DOI:** 10.1038/s41598-023-43184-w

**Published:** 2023-09-27

**Authors:** Bollen Martijn, Casaer Jim, Beenaerts Natalie, Neyens Thomas

**Affiliations:** 1 Centre for Environmental Sciences, UHasselt, Diepenbeek, Belgium; 2 Research Institute Nature and Forest, Brussels, Belgium; 3 Data Science Institute, UHasselt, Diepenbeek, Belgium; 4 Leuven Biostatistics and Statistical Bioinformatics Centre, KU Leuven, Leuven, Belgium

**Keywords:** Ecology, Ecological modelling

## Abstract

Knowledge on animal abundances is essential in ecology, but is complicated by low detectability of many species. This has led to a widespread use of hierarchical models (HMs) for species abundance, which are also commonly applied in the context of nature areas studied by camera traps (CTs). However, the best choice among these models is unclear, particularly based on how they perform in the face of complicating features of realistic populations, including: movements relative to sites, multiple detections of unmarked individuals within a single survey, and low detectability. We conducted a simulation-based comparison of three HMs (Royle-Nichols, binomial N-mixture and Poisson N-mixture model) by generating groups of unmarked individuals moving according to a bivariate Ornstein–Uhlenbeck process, monitored by CTs. Under a range of simulated scenarios, none of the HMs consistently yielded accurate abundances. Yet, the Poisson N-mixture model performed well when animals did move across sites, despite accidental double counting of individuals. Absolute abundances were better captured by Royle-Nichols and Poisson N-mixture models, while a binomial N-mixture model better estimated the actual number of individuals that used a site. The best performance of all HMs was observed when estimating relative trends in abundance, which were captured with similar accuracy across these models.

## Introduction

Biodiversity monitoring is increasingly important to improve our understanding of factors driving biodiversity changes. To keep up with the need for monitoring ecosystems globally, passive monitoring methods, including camera traps (CTs), have gained popularity^1,2^. In CT surveys, temporally replicated counts are collected at a set of geographical locations. From these counts, inference on animal abundance is possible, but it is typically complicated by imperfect detections and/or double counting^3^, especially when species are unmarked and individuals are therefore difficult to identify. This requires the use of statistical methods that separate process error, *i.e.,* variability in abundance, from observation error. Over the last decades, a wide variety of hierarchical models (HMs) have been developed for this aim (see Kéry and Royle^4,5^, for a recent synthesis). In these HMs, the data generating mechanism is represented by a mixture of distributions. The first distribution accounts for variation in the unknown abundance (representing the ecological process). Given the true abundance, the second distribution captures variation in repeated counts obtained from multiple, independent samples of the underlying abundance (representing the detection process). Two HMs that are frequently applied by ecologists are the Bernoulli-Poisson (BernP) mixture model^6^ (also called “Royle-Nichols model”) and the Binomial-Poisson (BP) mixture model^7^ (also called “Binomial N-mixture model”). The BernP and BP produce abundance estimates from, respectively, detection/non-detection data and count data collected at multiple locations (*e.g.* CT sites).

The problem with these models is that their parameter identifiability has been questioned^8^ (although Kéry ^9^ did not find identifiability issues for BP), and that they rely heavily on assumptions that are easily, and commonly, violated. BP and BernP assume that (i) the number of individuals present at a site does not change over the sampling period, commonly referred to as the closure assumption (note that we will refer to this assumption as “geographical closure” and to populations that satisfy/violate this assumption as “closed”/“open”), (ii) no false-positive detections occur, (iii) detections are independent of each other, and (iv) detection probability is equal among all individuals. Accidental double counting of individuals (a form of false positive detections) results in strong positive biases in BP^10^, but can, to some extent, be accommodated for by specifying a Poisson-Poisson (PP) mixture model (also called “Poisson N-mixture model”)^11^. Positive biases in BP also occur when detections are non-independent^12^. We are unaware of a study investigating the effect of unequal detection probabilities among individuals on the estimation quality of BP, but anticipate some degree of bias. Moreover, unmodelled heterogeneity in abundance and detection probability may also induce bias in abundances, particularly when data are sparse^10,13,14^.

But arguably one of the most common violations, which induces substantial bias in abundance, are departures from geographical closure^15^. For open populations, this bias in abundance occurs because individuals can potentially be detected at more than one sampling (*i.e.* CT) location. They are often dealt with by simply adjusting the interpretation of abundance to “frequency of site use”, the number of individuals that have used a site at least once (“superpopulation abundance” in Fogarty and Fleishman^15^). This interpretation of abundance acknowledges that individuals freely move across sites, analogous to “proportion of area occupied” in occupancy studies^16^. The classical conception of abundance, on the other hand, is closely tied to individuals remaining at their site (“season-long abundance” in Fogarty and Fleishman^15^). There are two problems with this. Firstly, site-use frequencies estimated from HMs will still suffer from other model violations (ii-iv), but it is unclear to what extent. Moreover, changing the interpretation of abundance does not fix the (claimed) problem of parameter identifiability. Relative trends in abundance (*i.e.* treating them as indices), on the other hand, should be identifiable^8,17^.

Hence, the objective of this study is to explore the estimator quality of three HMs for abundance of unmarked species (BernP, BP and PP) applied to CT data under a range of model violations, when abundance is viewed as site-use frequency and when abundances are regarded as indices (hereafter relative abundance). We therefore start by exploring the bias, root mean square error (RMSE) and 95% posterior credible interval (CI) coverage of traditional abundance estimates, and subsequently explore them for site-use frequency and relative abundance estimates. We consider various degrees of closure violations and double count frequency emerging from animal movement trajectories in a mid-sized nature area. It is our explicit choice to evaluate HMs not based on data generated by the analysis models, but based on CT detections (aggregated to counts) generated from a process that mimics the behaviour of wild boar, yet generic enough to apply to other mobile, group-living, unmarked species. This means that we simulate animal positions that are serially autocorrelated through a group-specific random walk movement model instead of assuming that their positions are realisations from a Poisson point process^11,18^. Inherently, this creates a mismatch between the data generating process assumed by HMs (*i.e.* a Poisson point process), and the actual process that generated the data. However, our strategy provides a realistic evaluation of how animal abundance estimation is typically approached in CT studies, which we believe is most useful to practitioners applying these models to their own CT data.

We show that PP, through accommodating for double counts, is able to estimate abundance more accurately compared to BP, and that interpreting estimates from HMs as frequency of site-use does not improve the inference in general, but leads to better estimates in BP. Finally, we report that while it is difficult to obtain accurate insight into absolute abundance, it is often possible to accurately estimate relative trends in abundance.

## Results

### Goodness-of-fit and predictive performance

The BernP had the highest leave-one-out (LOO) expected log-predictive density (ELPD) in each of the simulation scenarios (Supplementary fig. S1). The PP had a higher LOO-ELPD in all simulation scenarios, except for closed populations with a home range area (HRA) of 0.65 km^2^. All three HMs had Bayesian *P-values* that are close to zero across all simulations (Supplementary fig. S2).

### Accuracy and precision of parameter estimates

We assessed estimator quality by inspecting the relative bias, RMSE and 95% CI coverage for both observation and state parameters for each model under various combinations of study design, population size $N$ and HRA. Importantly, we found that increasing the number of grid cells that were sampled beyond 25% had a negligible impact on estimator quality (results not shown).

#### Detection parameters

**Figure 1:**
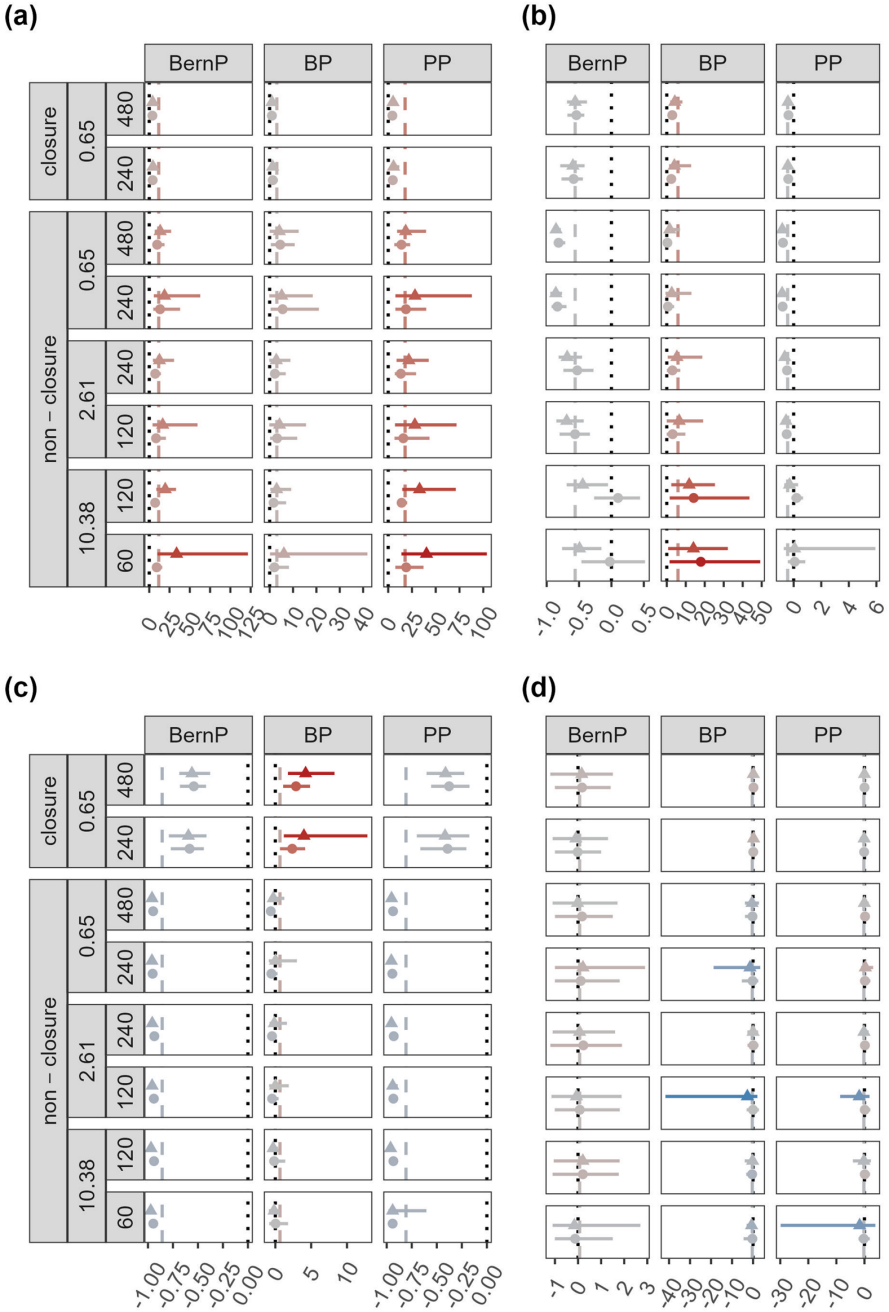
Mean relative bias (dots/ triangles), together with 2.5% and 97.5% quantiles (solid lines) in the estimated (**a**) detection parameters ${\theta }_{det}$, (**b**) abundances $\lambda$, (**c**) site-use frequencies ${\lambda }_{use}$ and (**d**) trends in $\lambda$. Results are displayed for all combinations of model used (BernP, BP, PP), population size N (60, 120, 240, 480), closure (closure, non-closure), movement parameters $\sigma$ (150, 300, 600, 1200) and $\rho$ (0.7: dots, 0.95: triangles), and the emerging home range area in km^2^ (0.65, 2.61, 10.38). Line of equality (dotted line). Average relative bias for each HM (dashed line). For visual clarity, x-scales are different for the subpanels in (**b**) and (**c**).

**Figure 2:**
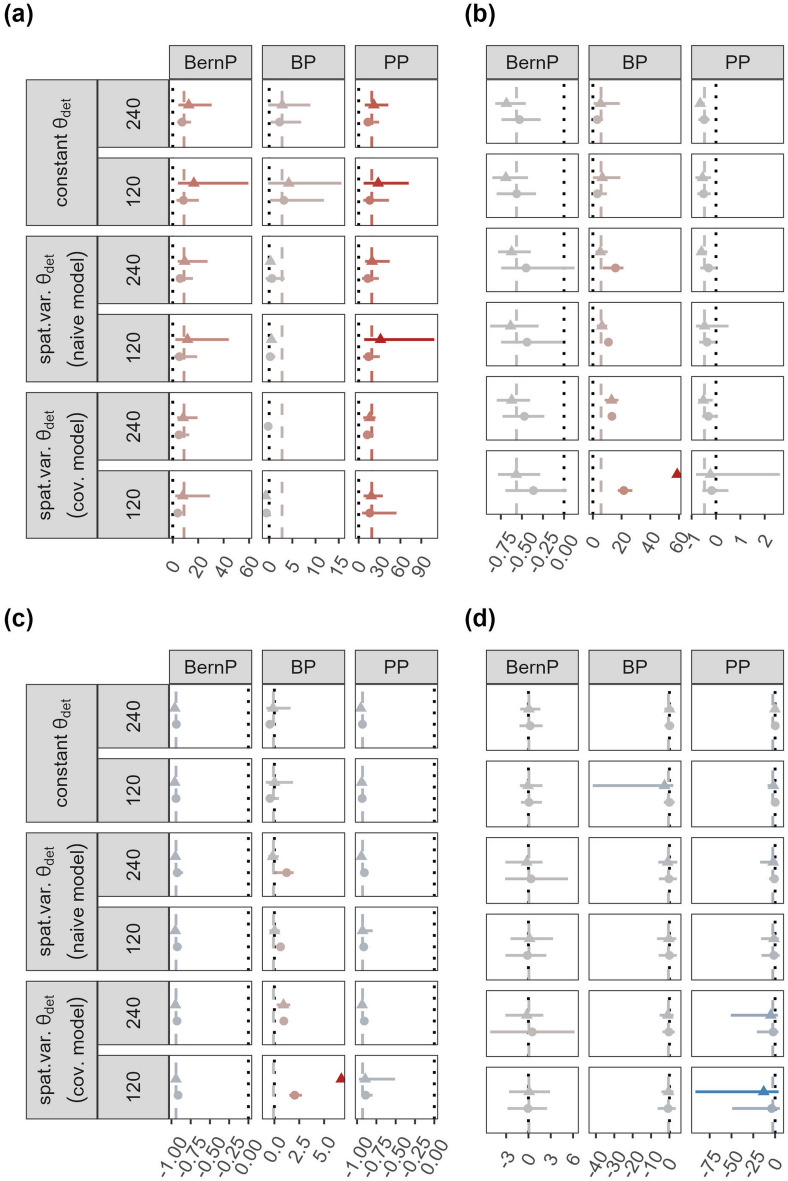
Mean relative bias (dots/ triangles), together with 2.5% and 97.5% quantiles (solid lines) in the estimated (**a**) detection parameters ${\theta }_{det}$, (**b**) abundances $\lambda$, (**c**) site-use frequencies ${\lambda }_{use}$ and (**d**) trends in $\lambda$. Results are displayed for all combinations of model used (BernP, BP, PP), the submodel and covariate structure for ${\theta }_{det}$ (constant, spatial variation (naïve model), spatial variation (covariate model)), population size N (120, 240), $\rho$ (0.7: dots, 0.95: triangles) for $\sigma$ = 300 (emerging home range area of 2.61 km^2^). Line of equality (dotted line). Average relative bias for each HM (dashed line). For visual clarity, x-scales are different for the subpanels in (**b**) and (**c**).

All HMs consistently overestimated ${\theta }_{det}$, and the relative bias increased as the HRA increased (Fig. 1a; Table 1). Furthermore, none of the models reached the 95% CI coverage threshold for any of the simulation scenarios. The BP is the only model that reached coverages > 10 in most of the scenarios (Supplementary table S1). Nevertheless, the RMSE for BernP and BP is relatively small, with median values across all scenarios around 0.05 and 0.02 respectively. Overall, ${\widehat{\theta }}_{det}$ obtained through the BP were the most accurate according to the median proportion of simulations with |Relative Bias|≤ 0.5 (Table 1). For BernP and PP, patterns in relative bias were similar for constant ${\theta }_{det}$, ${\theta }_{det}$ with spatial variation analysed by naïve and covariate models (Fig. 2a). However, for BP the relative bias in ${\theta }_{det}$ decreased when spatial variation was present. In addition, these scenarios led to lower variability in relative bias in ${\theta }_{det}$.

**Table 1:** Summary table for estimator quality of detection parameters ${\theta }_{det}$, abundances $\lambda$ and site use frequencies ${\lambda }_{use}$ obtained from three Bayesian hierarchical models (BernP, BP, PP).

HRA (km^2^)	N	$\rho$	|Relative Bias|≤ 0.5								
			Detection parameters ${\theta }_{det}$			Abundance $\lambda$			Site use frequency ${\lambda }_{use}$		
			BernP	BP	PP	BernP	BP	PP	BernP	BP	PP
0.65 (closure)	480	0.70	0.00	0.25	0.00	0.30	0.00	0.90	0.30	0.00	0.90
		0.95	0.00	0.25	0.00	0.28	0.00	0.83	0.28	0.00	0.83
	240	0.70	0.00	0.15	0.00	0.15	0.03	0.83	0.15	0.03	0.83
		0.95	0.00	0.15	0.00	0.13	0.00	0.73	0.13	0.00	0.73
0.65	480	0.70	0.00	0.03	0.00	0.00	0.73	0.00	0.00	0.18	0.00
		0.95	0.00	0.15	0.00	0.00	0.43	0.00	0.00	0.33	0.00
	240	0.70	0.00	0.05	0.00	0.00	0.55	0.00	0.00	0.25	0.00
		0.95	0.00	0.10	0.00	0.00	0.25	0.00	0.00	0.38	0.00
2.61	240	0.70	0.00	0.08	0.00	0.38	0.08	0.58	0.00	0.48	0.00
		0.95	0.00	0.20	0.00	0.05	0.08	0.08	0.00	0.45	0.00
	120	0.70	0.00	0.05	0.00	0.38	0.15	0.48	0.00	0.43	0.00
		0.95	0.00	0.20	0.00	0.10	0.13	0.13	0.00	0.30	0.03
10.38	120	0.70	0.00	0.33	0.00	0.98	0.00	0.93	0.00	0.50	0.00
		0.95	0.00	0.10	0.00	0.60	0.00	0.65	0.00	0.70	0.00
	60	0.70	0.00	0.35	0.00	0.93	0.03	0.85	0.00	0.33	0.00
		0.95	0.00	0.13	0.00	0.48	0.03	0.63	0.00	0.63	0.00
41.58	60	0.70	0.00	0.30	0.00	0.15	0.00	0.08	0.00	0.58	0.00
		0.95	0.00	0.03	0.00	0.85	0.00	0.75	0.00	0.50	0.00
	30	0.70	0.00	0.35	0.00	0.13	0.00	0.15	0.00	0.50	0.00
		0.95	0.00	0.03	0.00	0.75	0.00	0.58	0.00	0.55	0.00
Median			0.00	0.15	0.00	0.21	0.03	0.58	0.00	0.40	0.00

Cells display the proportion of simulation replicates that satisfy |Relative Bias|≤ 0.5.

#### Abundance

Regardless of the simulation scenario, BP consistently overestimated abundance $\lambda$ (Fig. 1b). Both BernP and PP slightly underestimated $\lambda$ in most of the simulation scenarios. Overall, the relative bias in $\lambda$ was nearly unaffected by the HRA. However, the relative bias in $\lambda$ increased for BP, and decreased for BernP and PP when population sizes $N$ become smaller. When HRA ≥ 10.38 and ρ = 0.70, 95% CI coverages were close to the threshold for BernP and PP (Supplementary table S2). None of the 95% CI coverages in BP reached the threshold. Furthermore, RMSEs were generally low for BernP and PP, but not for BP. According to the median, PP most accurately estimated $\lambda$, followed by the BernP and BP, with respectively 58%, 21% and 3% of simulations resulting in |Relative Bias|≤ 0.5 (Table 1). Positive bias in $\lambda$ from BP was stronger when spatial variation in ${\theta }_{det}$ was present, especially when this information was used in a covariate model (Fig. 2b). For all models, the variability in relative bias in $\lambda$ lacked a clear pattern.

#### Site-use frequency

For open populations, relative bias in ${\lambda }_{use}$ differs considerably with that observed for $\lambda$ for all models (Fig. 1c). Both BernP and PP strongly underestimated ${\lambda }_{use}$ in all simulation scenarios, while BP slightly underestimated or overestimated ${\lambda }_{use}$ depending on the simulation scenario (Fig. 1c). Negative bias in ${\lambda }_{use}$ most frequently occurs when ρ = 0.70, while positive bias dominated when ρ = 0.95. Negative bias in ${\lambda }_{use}$ was observed for BernP and PP for all simulation scenarios. None of the HMs was able to reach the 95% CI coverage threshold in any of the simulation scenarios, and even for the most accurate model, *i.e.*, BP, the median RMSE across all simulations was substantial (Supplementary table S3). According to the median proportion of simulations resulting in |Relative Bias|≤ 0.5, BP accurately estimated ${\lambda }_{use}$ in half of the cases, while both PP and BP failed to produce accurate estimates of ${\lambda }_{use}$ (Table 1). The patterns in relative bias in ${\lambda }_{use}$ were affected by spatial variation in ${\theta }_{det}$ in a similar way as those in $\lambda$, that is, stronger positive relative bias for BP (Fig. 2c).

#### Relative abundance trends

When relative trends in abundance $\lambda$ were of prime interest, BernP and PP outperform BP in nearly all scenarios (Table 2). Despite some variability in accuracy, Fig. 1d revealed that the mean estimated trends in $\lambda$ were close to the true trends for all models and for all simulation scenarios. However, the |Relative Bias|≤ 0.5 revealed a tendency for poorer trend estimates with stronger departures from closure. According to the median, the models yielded respectively 52% and 69% (BernP), 44% and 56% (BP), and 50% and 65% (PP) of trend estimates (10% and 20% decline in population size $N$) with a |Relative Bias|≤ 0.5 (Table 2). Average relative bias in trend estimates were nearly unaffected by spatial variation in ${\theta }_{det}$ (Fig. 2d). However, the variability in trend estimates from BernP and PP, but not BP, was substantially larger when spatial variation in ${\theta }_{det}$ was present. Finally, trend estimates from this scenario had a lower accuracy across all these models (Supplementary table S5).

**Table 2:** Summary table for estimator quality of relative abundance (10% trend: ${\lambda }_{N}/{\lambda }_{0.9N}$ and 20% trend: ${\lambda }_{N}/{\lambda }_{0.8N}$) obtained from three Bayesian hierarchical models (BernP, BP, PP).

HRA (km^2^)	N	$\rho$	|Relative Bias|≤ 0.5					
			10% trend			20% trend		
			BernP	BP	PP	BernP	BP	PP
0.65 (closure)	480	0.70	0.66	0.76	0.75	0.83	0.88	0.87
		0.95	0.64	0.53	0.72	0.84	0.78	0.88
	240	0.70	0.74	0.81	0.62	0.90	0.95	0.83
		0.95	0.61	0.67	0.56	0.84	0.84	0.83
0.65	480	0.70	0.53	0.56	0.58	0.82	0.81	0.78
		0.95	0.52	0.47	0.55	0.73	0.63	0.77
	240	0.70	0.47	0.48	0.49	0.69	0.69	0.65
		0.95	0.42	0.36	0.42	0.66	0.48	0.63
2.61	240	0.70	0.53	0.49	0.53	0.73	0.73	0.72
		0.95	0.62	0.36	0.60	0.78	0.58	0.70
	120	0.70	0.58	0.56	0.52	0.71	0.53	0.66
		0.95	0.37	0.36	0.42	0.68	0.42	0.64
10.38	120	0.70	0.54	0.34	0.53	0.62	0.53	0.63
		0.95	0.50	0.43	0.39	0.63	0.44	0.56
	60	0.70	0.46	0.45	0.39	0.68	0.58	0.59
		0.95	0.31	0.34	0.26	0.52	0.39	0.39
41.58	60	0.70	0.53	0.31	0.45	0.61	0.47	0.58
		0.95	0.46	0.37	0.38	0.62	0.50	0.51
	30	0.70	0.42	0.37	0.29	0.58	0.42	0.46
		0.95	0.32	0.37	0.19	0.52	0.45	0.34
Median			0.52	0.44	0.50	0.69	0.56	0.65

Cells display the proportion of simulation replicates that satisfy |Relative Bias|≤ 0.5.

## Discussion

In this study, we have evaluated three hierarchical abundance estimators (BernP, BP and PP) in the context of complicating features of realistic animal populations simulated through random walks.

Increasing trends in per-capita detectability and detection rate with population size $N$, as indicated in Table 3**,** are within the line of expectations, since denser populations are expected to result in more frequent detection events (*i.e.*, an individual crossing the viewshed). We follow Neilson, et al.^18^, in assuming that populations of individuals occupying large HRAs, such as apex predators, are generally less dense (Table 3). This assumed confounding of HRA and population size, explains the decreasing trend of ${\theta }_{det}$ with HRA**.** Evidently, the per-capita detection probability is consistently lower than the per-capita detection rate, as some individuals may be detected more than once on a survey day. Regardless of geographical closure and the HRA, the abundance $\lambda$ equals $N/{N}_{site}$, resulting in the densities indicated in Table 3 (“Abun.”). Site-use frequency, ${\lambda }_{use}$, is a product of both population size $N$ and HRA. In theory, ${\lambda }_{use}$ increases with both increasing $N$ and HRA. However, in our simulations these parameters are confounded to simulate realistic animal populations^18^. Hence, ${\lambda }_{use}$ is seemingly unaffected by $N$ and HRA, as their effects cancel out. Only, the effect of closure violations on ${\lambda }_{use}$ is clearly visible (Table 3; “Site-use freq.”).

**Table 3:** Design of the simulation study, based on three ecological/ behavioural parameters, home range area, population size and mean hourly displacement of (a group of) individual animals.

Home range		Population size			Movement		Detection process	
Closure	HRA	N	Abun. ($\lambda )$	Site-use freq. (${\lambda }_{use})$	$\sigma$	$\rho$	$p$	$\mu$
Closure	0.65	480	3.33	3.33	150	0.70	0.0297–0.0554	0.0411–0.0831
						0.95	0.0158–0.0380	0.0322–0.0749
		240	1.67	1.67		0.70	0.0221–0.0638	0.0288–0.0955
						0.95	0.0129–0.0411	0.0249–0.0879
Non-closure		480	3.33	11.49 – 15.88		0.70	0.0036–0.0192	0.0049–0.0274
						0.95	0.0019–0.0136	0.0029–0.0252
		240	1.67	5.58 – 7.94		0.70	0.0018–0.0191	0.0025–0.0321
						0.95	0.0011–0.0148	0.0015–0.0351
	2.61	240	1.67	11.82 – 13.14	300	0.70	0.0024–0.0155	0.0030–0.0195
						0.95	0.0009–0.0090	0.0014–0.0132
		120	0.83	5.69 – 6.78		0.70	0.0017–0.0157	0.0019–0.0177
						0.95	0.0005–0.0125	0.0011–0.0179
	10.38	120	0.83	14.09 – 16.48	600	0.70	0.0036–0.0094	0.0042–0.0109
						0.95	0.0011–0.0044	0.0013–0.0051
		60	0.42	6.79 – 8.62		0.70	0.0027–0.0107	0.0027–0.0124
						0.95	0.0002–0.0057	0.0002–0.0068
	41.58	60	0.42	17.44 – 22.84	1200	0.70	0.0019–0.0059	0.0019–0.0065
						0.95	0.0003–0.0041	0.0005–0.0054
		30	0.21	8.11 – 11.84		0.70	0.0015–0.0067	0.0015–0.0071
						0.95	0.0001–0.0044	0.0004–0.0056

The range of true detection probabilities $p$ and rates $\mu$, resulting from each scenario, are also indicated.

In our simulations, true ${\theta }_{det}$ are extremely small (*i.e.,* always ${\theta }_{det}$ < 0.05). Hence, it is perhaps not surprising that in these scenarios positive bias in ${\theta }_{det}$ persisted across most of the HMs considered (Figs. 1a and 2a). It is notoriously difficult for these HMs to accurately estimate parameters near the boundary of their parameter space^20,21^. In cases where ${\theta }_{det}$ is accurately estimated, abundance estimates appear to suffer from increased positive bias (Fig. 2a–c BP—covariate model) in order to provide an explanation for the observed counts (*i.e*. some incidences of high counts occur because of accidental double counting). The use of a beta-binomial distribution for $p$ in the BP case, which can alleviate bias resulting from correlated detections within samples, may alleviate some of this bias in abundance, while conserving accuracy in ${\theta }_{det}$ ^12^. Overall, we would like to warn practitioners that very small detection probabilities/ detection rates, which are frequent in camera trapping studies, often do not contain sufficient information to confidently analyse within complex modelling frameworks. However, modelling detections of multiple species jointly may hold the potential to overcome issues related to low detectability^22^.

Furthermore, our results reveal that none of the HMs considered in this study were able to estimate $\lambda$ accurately under all scenarios (Figs. 1b and 2b). The BernP model slightly underestimates $\lambda$ in most scenarios as opposed to simulation results of Royle and Nichols^6^. Nonetheless, BernP estimates $\lambda$ more accurately than BP, despite only using binary input data. A possible explanation for the binary input model to outperform a count model lies in the occurrence of double counting of individuals. Indeed, we assumed that animals were unmarked, and hence could not be individually identified by CTs, which led to badly inflated ${\widehat{\lambda }}^{BP}$, a well-known issue for BP^10,13^. However, this problem does not apply to binary models, since double counts do not affect a binary encoding. PP, which alleviates most of the bias in $\lambda$ observed under BP, produces the most accurate estimates of $\lambda$ from any of the models considered here. In line with our expectations, a PP even yields accurate estimates in over 80% of the cases when populations are closed (Table 1). This highlights the importance of accounting for double counts when absolute abundances are desired. Nonetheless, ${\widehat{\lambda }}^{PP}$ are still somewhat negatively biased in open populations. Specifying a distribution for the latent ${\mathrm{\rm N}}_{i}$’s that allows for overdispersion and/or underdispersion can be a solution to improve inference when extra-variation in counts is present among sites. We have used zero-inflated Poisson distribution for ${\mathrm{\rm N}}_{i}$’s^19^, which did not result in more accurate $\lambda$’s (results not shown). On the contrary, both ${\widehat{\lambda }}^{BZIP}$ and ${\widehat{\lambda }}^{PZIP}$ display some of the strongest biases that we observed in our simulations. This is likely a consequence of these models being very complex, *i.e.*, they require the estimation of two latent parameters in addition to a detection probability/ detection rate, for the sparse data simulated in our study. Despite their inaccurate estimates BZIP and PZIP models were preferred by LOO-ELPDs, which is possibly because they provide a good description of the many zero-observations present in the data^3,19^. Negative binomial models have also been suggested to deal with extra-variation in counts, but suffer from a similar “good-fit-bad-prediction dilemma”^4,23^. Hence, there seems no reason to assume that HMs with negative binomial state models perform any better than the zero-inflated Poisson HMs evaluated here.

Given that $\lambda$ is not well estimated for open populations, the use of HMs for inferring the site-use frequency ${\lambda }_{use}$ could be an alternative that provides reliable answers to many wildlife related questions. Site-use frequency is effectively a product of population size and HRA, hence reflects both the true number ($N$) of animals present, as well as their movement pattern^11,15,24^. We expected that this quantity (${\lambda }_{use}$) would be better estimated than abundance ($\lambda$) when populations are open, yet our results suggest that this is not the case (Figs. 1b–c and 2b–c). The BernP, which uses only binary input, is too conservative to produce estimates in the order of magnitude of ${\lambda }_{use}$. PP on the other hand can attribute additional counts resulting from groups moving across sites to a higher detection rate ($\mu$). This seems to be supported by estimates of detection rates in the PP that steadily increase with increasing HRA (Fig. 1a). Site-use frequency estimated from BP outperform those of BernP and BP, but, on average, they still only yield accurate results in 40% of the simulations. Contrasting with our results, Nakashima^11^ found that BernP and PP, and not BP, were able to accurately estimate the site-use frequency. Fogarty and Fleishman^15^, on the other hand, also find that a BP, which accounts for permanent movements among sites (*i.e.*, immigration and emigration) yields accurate estimates of site-use frequency (“superpopulation abundance”). However, they point out that the ecological interpretation of site-use frequency is difficult when the movement characteristics of animals are unknown. The problem is that there is no way of discriminating between sites that are frequently used because they are on a travelling route and between sites that are frequently used because they offer important resources. Thus, site-use frequency does not appear to provide a reliable solution for the interpretation of abundances of population that are open relative to the sampling frame. Abundance (or density) of open populations may be estimated more accurately by spatially-explicit models for count data, which leverage observed spatial correlation in detections^25^. However, for entirely unmarked populations, which we simulated in this study, the precision of abundance/density estimates based on this method is typically low^26,27^. Therefore, future developments in spatially-explicit models for count data should focus on improving precision of their estimates.

Regardless of the capability of HMs to yield unbiased estimates of $\lambda$ (or ${\lambda }_{use}$), it is often more interesting to explore relative trends in $\lambda$. Moreover, while there are concerns regarding identifiability within HMs for abundance, their ability to estimate relative abundances has not been questioned^8,17^. Here, we explored trend estimates obtained through comparing abundances ${\lambda }_{N}/{\lambda }_{0.9N}$ and ${\lambda }_{N}/{\lambda }_{0.8N}$ to induced declines of respectively, 10 and 20%. Overall, simulated declines in population size $N$ of 10 and 20%, are accurately estimated more frequently than standalone $\lambda$ (Table 2). However, when a relative bias up to 50% is tolerated, 48%, 66%, 50% and 31%, 44%, 35% of the trends will be misleading for respectively 10% and 20% declines estimated by BernP, BP and PP. Thus, HMs for abundance are better suited to capture relative trends in abundance, but obtaining them post-hoc, as is often done by practitioners, may still be misleading in many cases. Estimates of relative trends in abundance may be biased particularly when they are confounded with between-year variation in detection probability that is unaccounted for. Because of our focus on a single sampling season within a single year, we did not evaluate the impact of temporal variation in the probability of detecting an individual that crosses a camera viewshed, *i.e.,* P(detected|in viewshed), which is a study limitation. However, the true detection probability is a product of P(detected|in viewshed) and the probability that an individual crosses a camera viewshed if it is present, *i.e.*, P(in viewshed). Inherent variation in the simulated movement trajectories of our study induce small-scale variation in P(in viewshed), and from our results, it appears that relative trends are robust against this form of variation. Our results suggest that when spatial variation in P(detected|in viewshed) exists, the accuracy of relative trends from BernP, BP and PP decrease owing to a larger variability (Fig. 2d; supplementary table S5). Future research should determine if relative trends in abundance suffer from similar losses in accuracy when stronger temporal variation in detection probability exist.

While there are potentially many other candidate models that are useful to estimate animal abundance (or density) in ecological surveys, we explicitly chose not to include them in our comparison. We did not include distance sampling^28^ or random encounter (and staying time) models^29,30^ as they are hampered by a need for additional information, either distance data or movement data that are often absent or of low quality in camera trapping studies. We did not consider capture-recapture methods^31^ since identifying individuals through photographs is difficult when individuals are unmarked, which was one of the assumptions of our study. For the same reason, we disregarded methods based on removal sampling^32,33^, where individuals need to be identified to be removed from the study after their first detection. Finally, we did not include models in Moeller, et al.^34^: the time-to-event model was disregarded because it assumes perfect detectability, the space-to-event and instantaneous sampling models require time-lapse photos which may lead to many missed encounters.

In conclusion, we have shown that under realistic settings, and with 24-h as the time unit of temporal replicates, the probabilities of detecting an unmarked species like wild boar using camera traps are extremely low. In addition, when individuals cannot be individually identified, and thus double counting cannot be excluded, we find strong biases in estimates of abundances $\lambda$ for BP. Through accommodating false-positives, the PP model was able to estimate $\lambda$ more accurately. Furthermore, we reveal that shifting the interpretation to frequency of site-use does not improve the inference in general, but leads to better estimates in BP. Finally, we report that relative trends in abundance are estimated with greater precision than absolute abundances, but that they can still suffer from bias especially when spatial variation in detection probability exist. Practitioners should thus avoid using absolute abundance estimates, regardless of their interpretation, from static HMs and turn to relative trend estimates, but also these should be used with caution. Depending on the context, model-based approaches, taking into account temporal trends, spatial trends or a combination thereof, might further improve the accuracy of inference from HMs. From this perspective, it would be valuable to assess the estimator quality of temporally explicit HMs in a simulation study similar to ours.

## Material and methods

Our simulation study is designed with a specific unmarked target species (wild boar) in mind, in a specific park (Hoge Kempen National Park (NPHK) situated in Belgium). In that park, the species commonly investigated throughout a relatively short survey period within a single year, a strategy we adopt as well. Yet, our findings, based on this design, are applicable to a wider range of camera trapping studies since most of them take place in areas of comparable size, with similar CT spacing and with a focus on mammal species, many of which live in groups and display home range tendencies^1,35^. In fact, ungulates comprised the second most studied group of species according to Burton, et al^1^. Moreover, we do not simulate climatic conditions or landscape features that are specific to the NPHK.

### Simulated space

**Figure 3:**
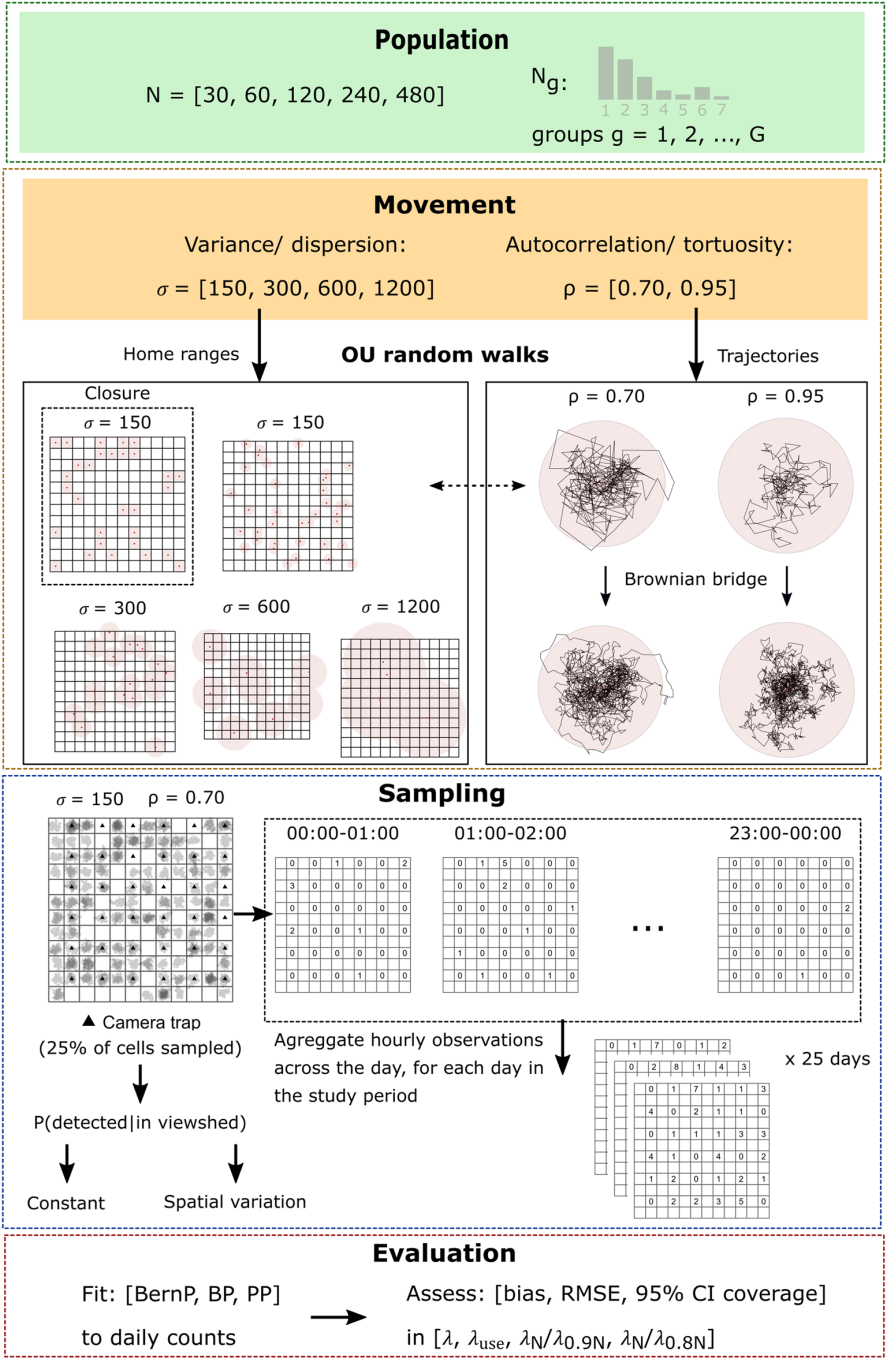
Graphical overview of the simulation study. The dimensions of the state space represented by the grid layers are 10.8 km × 10.8 km. OU: Ornstein–Uhlenbeck, BernP: Bernoulli-Poisson mixture model, BP: Binomial-Poisson mixture model, PP: Poisson-Poisson mixture model.

We simulated a space with a total surface area of 116.64 km^2^ (10.8 km * 10.8 km; Fig. 3), which roughly equals that of the NPHK^36^. Next, we simulated a grid layer consisting of 144 grid cells ($i = 1, 2, \dots , 144$) of 0.9 km × 0.9 km, for the placement of CTs. Our grid cells are nine times coarser than the grid cells in Wevers, et al.^36^, such that the area (0.81 km^2^) of a single grid is closer to the empirical home range sizes of wild boar^37,38^. Next, we placed a CT in 25% (36) of the grid cells, according to a randomised regularly spaced sampling design. Within sampled grid cells, a CT “detector” was then simulated by placing a single CT at its centroid with a viewshed radius $r = 15 m$ and an angle of view $\theta = 42^\circ$, based on camera specifications of the Reconyx Hyperfire HC600^39^. All CT viewsheds, in which passing animals may be detected, were simulated facing North.

### Movement trajectories

We assumed that $N$ individuals of a species of interest (with $N$ fixed a priori according to Table 3**)** live in the region of interest, say $A$. Moreover, we assumed that these animals moved in groups, which we monitored during 25 consecutive days within a single season in which environmental factors potentially affecting probabilities of detecting an individual were assumed to be more or less constant over time. We started by simulating forty independent, group-specific and activity-adjusted random walks according to a bivariate Ornstein–Uhlenbeck process (OU). To generate realistic animal trajectories for a species with home range tendencies, we varied three parameters: the population size ($N$) and parameters that control serial autocorrelation ($\uprho$) and variance in movements ($\upsigma$) (Table 3). Note that the size of the HRA emerges from the choice of $\upsigma$.

Let $u$ represent a point location, defined by a set of coordinates $\{x,y\}$. For $g = 1, \dots , G$ groups and $t = 1, \dots , 600$ one-hour steps (25 days of 24 h), the probability for a given set of group-specific positions ${{\varvec{u}}}_{{\varvec{g}}({\varvec{t}}+1)}$ in the next hour $t+1$ according to a discrete-time OU can be expressed by:

$$p\left({{\varvec{u}}}_{{\varvec{g}}\left({\varvec{t}}+1\right)}|{{\varvec{u}}}_{gt},{{\varvec{s}}}_{{\varvec{g}}}\right)=Norm\left({{\varvec{s}}}_{{\varvec{g}}}+{e}^{\rho {\varvec{I}}}\left({{\varvec{u}}}_{gt}-{{\varvec{s}}}_{{\varvec{g}}}\right),{\sigma }^{2}{\varvec{I}}-({e}^{\rho {\varvec{I}}}\cdot {\sigma }^{2}{\varvec{I}}\cdot {e}^{\rho {\varvec{I}}})\right)$$

where ${{\varvec{s}}}_{{\varvec{g}}}$ are the group-specific home range centers,$\rho$ controls the tendency to move in the direction of the home range center, $\sigma$ determines the diffusion of the movement and ${\varvec{I}}$ represents the 2 × 2 identity matrix. However, when the species was resting (here we assumed a nocturnal species, but the results would be similar for a diurnal species with similar proportions of active time), we assumed that all groups stayed in place, hence $$\begin{array}{cc}{{\varvec{u}}}_{{\varvec{g}}\left({\varvec{t}}+1\right)}={{\varvec{u}}}_{{\varvec{g}}{\varvec{t}}}& if (t+1) \% 24 \in \{0:5, 18:23\}\end{array}$$, where ‘%’ represents the modulo.

We generated group sizes ${N}_{g}$ for each group $g$ by sampling empirical group sizes of the target population that satisfy $N= {\sum }_{g=1}^{G}{N}_{g}$. In addition, we simulated 10% and 20% declines in population size for all populations (i.e., ${N}_{0.9}=0.9N$ and ${N}_{0.8}=0.8N$) to explore the ability of the models to estimate these declines by comparing abundances *post-hoc* (${\lambda }_{N}/{\lambda }_{0.9N}$ and ${\lambda }_{N}/{\lambda }_{0.8N}$). Group-specific home range centers ${{\varvec{s}}}_{{\varvec{g}}}$ were (i) sampled from the fixed set of 0.9 km × 0.9 km grid centroids (resulting in closure) or (ii) sampled randomly from the entire study space (resulting in closure violations). When simulating hourly displacements, information on finer scale movements is lost. However, these may be important when they result in the passing or not passing through the camera viewshed^40^. To ensure that we generated more realistic, finer scale movements, we simulated Brownian motion between consecutive positions ${x\boldsymbol{ }={\varvec{u}}}_{gt}$ and ${y={\varvec{u}}}_{g(t+1)}$ using a Brownian Bridge (Supplementary section 1).

### Simulating count and detection-non detection data

After simulating animal movements, we generated sightings for each hour $h = 1, 2, ..., 24$ within each day $j = \mathrm{1,2},\dots , 25$ at locations where a group of individuals crossed the CT viewshed, for $i=1, 2,\dots , 36$ CT locations. We assumed that all individuals in a group were detected when they pass this viewshed (*i.e.,* P(detected|in viewshed) = 1). Note that the probability to detect an individual also depends on the probability that an individual that is present enters a CT viewshed (*i.e.,* P(in viewshed), which will be <  < 1). Additionally, we assessed the impact of imperfect P(detected|in viewshed) ≈ 2/3, which also display spatial variation according to a unique Matérn process^41^ per simulation for a subset of the simulation scenarios (*i.e.,* σ = 300 resulting in HRA = 2.61 km^2^) that best reflect empirical home range areas of the target species. In both cases, we obtained hourly counts as*: *${y}_{ijh} \sim binomial({n}_{ijh}, {P[det|in view]}_{i})$*,* where ${n}_{ijh}$ is the number of individuals crossing a CT viewshed located at grid cell $i$ on day $j$ and hour $h$. Hourly counts ${y}_{ijh}$ were then aggregated across days to yield daily summaries of counts ${y}_{ij}=\sum_{h=1}^{24}{y}_{ijh}$. Since we have assumed that individuals are unmarked, the same group could potentially contribute to ${y}_{ij}$ on each hour if they re-appeared in front of the CT. When only binary inputs were required (*i.e.* for BernP), we reduced count data to detection/non-detection data.

### Assessment of criteria for the model inferences

For each simulation replicate, we calculated the true abundance ($\lambda$) as the average number of individuals per grid cell, assuming that all individuals are bounded to the grid cells that contain their activity centers (*i.e.,* the average number of activity centers per grid). This quantity was used as ground truth to assess the ability of the HMs to make accurate inferences on $N$. In simulation scenarios with closure violations, individuals move across their initial grid cells such that the number of individuals per grid cell changes over time. Hence, we also calculated site-use frequency (${\lambda }_{use}$) as the average number of individuals that have used a grid cell at least once during the simulated study period, given that all individuals completely covered their HRA (*i.e.,* the average number of HRAs overlapping a grid cell)^11,15^. Finally, we assessed the ability of HMs to accurately estimate relative abundances by comparing how accurately ${\lambda }_{N}/{\lambda }_{0.9N}$ and ${\lambda }_{N}/{\lambda }_{0.8N}$ captured true induced population declines of respectively 10 and 20% for different choices of $N$.

### Statistical models

Within the current study, we assessed the performance of HMs, that are commonly used to make inference on abundance. Depending on the data at hand, BernP^6^ (for replicated detection/ non-detection data) and BP/PP^7,11^ (for replicated count data) models are commonly used. In this study, we adopt a Bayesian estimation framework, as it allows the flexible modelling of HMs. Model fitting was performed using *Stan* (via the R package *cmdstanr*), a probabilistic programming language that enables Bayesian estimation through a dynamic Hamiltonian Monte Carlo sampler^42^.

All three statistical models start from the series of observations ${y}_{ij}$. Observations ${y}_{ij}$ can either consist of binary detection ${(y}_{ij}=1)$/ non-detection ${(y}_{ij}=0)$ data or count ${(y}_{ij}\ge 0)$ data. The mathematical structure and distributional assumptions for ${y}_{ij}$ and ${N}_{i}$, the number of individuals present at site $i$, are given in Table 4. Note that in practice, ${N}_{i}$’s are obtained by taking the finite sum over $K$ possible latent abundances to make the maximisation of the likelihood numerically tractable^7^. Here we chose an upper bound $K$, such that $K=100$ for detection/ non-detection data and $K = max\left({y}_{ij}\right)+100, \forall \{i,j\}$ for count data. Moreover, we construct the likelihood by marginalising over ${N}_{i}$’s with upper bound $K$ given that *Stan* cannot sample discrete latent variables. Importantly, $p$ – the per capita detection probability – represents the probability that an individual is detected (assuming that there are no false-positives), while $\mu$ – the per capita detection rate – corresponds to the rate at individuals are detected each day (when false-positives occur). For notational simplicity, we will refer to these detection parameters jointly as ${\theta }_{det}$. For simulation runs with spatial variation in P(detected|in viewshed), the ‘naïve’ models as in Table 4 were fitted alongside ‘covariate’ models that used the values from the Matérn process, which generated P(detected|in viewshed), as a covariate $x$: $logit({\theta }_{det})={\beta }_{0}+{\beta }_{1}x$*.*

**Table 4:** Mathematical structure of the hierarchical models used in this study, their main reference and applications in wildlife research.

Model	Miaxture	Data	Observation model	State model	Ref	Sample of applications
BernP	Bernoulli/Poisson	Binary	$P=1-{\left(1-p\right)}^{{N}_{i}}$ ${y}_{ij}|{N}_{i}\sim Bernoulli(P)$	${N}_{i}\sim Poisson\left(\lambda \right)$	^6^	^45,46^
BP	Binomial/Poisson	Counts	${y}_{ij}|{N}_{i}\sim Binomial({N}_{i}, p)$		^7^	^47,48^
PP	Poisson/Poisson		${y}_{ij}|{N}_{i}\sim Poisson({N}_{i}\cdot \mu )$		^11^	^49^

To assess goodness of fit for competing HMs, we calculated Bayesian *P*-values^10,43^. Additionally, we computed and compared the LOO-ELPD for each model^44^. For more information on the prior specification, goodness-of-fit evaluation and MCMC convergence of HMs in Stan we refer to Supplementary sections 2–3. To give a representative view on the results, we did not exclude simulation runs where convergence and/or identifiability issues occurred from further analysis.

### Relative bias, RMSE and coverage probability

In this study, we assessed estimator performance from three Bayesian HMs of interest by exploring the bias, relative bias, RMSE and 95% CI coverage. We calculated the bias as $Bias\left(\theta \right)= (\widehat{\theta }-\theta )$; the relative bias as $Rel. Bias\left(\theta \right)= (\widehat{\theta }-\theta )/\theta$; the $RMSE\left(\theta \right) = \sqrt{{\sum }_{i=1}^{n}{({\widehat{\theta }}_{i}-{\theta }_{i})}^{2}/n}$); and the 95% CI coverage as the proportion of simulations where the true parameter value is enclosed by the 95% CI. We used |relative bias|< 0.5 as the threshold for simulations that yielded accurate estimates. Moreover, we regard 0.95 of simulations that reached the 95% CI coverage as an indicator for good uncertainty quantification.

### Supplementary Information

Supplementary Information.

## Data Availability

All the data used here is available in Supplementary Information (Supplementary Data) and at https://github.com/MartijnUH/RWsim_abundance_models.
