# Supplementary material for: Simulation-based assessment of the performance of hierarchical abundance estimators for camera trap surveys of unmarked species
Source: Sci Rep. 2023 Sep 27;13:16169. doi: 10.1038/s41598-023-43184-w (PMC10533874; doi:10.1038/s41598-023-43184-w)
Supplement: Supplementary file 1 — Supplementary Information. [file 41598_2023_43184_MOESM1_ESM.pdf]

Supplementary materials for "Simulation-based assessment of the performance of hierarchical abundance estimators for camera trap surveys of unmarked species"

Bollen Martijn, Casaer Jim, Beenaerts Natalie and Neyens Thomas

1 Mathematical description of the Brownian Bridge

The movement model explained in the Material and Methods section of the main paper, simulates group-specific movements at hourly intervals. However, to make the animal trajectories generated from our model more realistic, we added Brownian motion to each of the line segments representing hourly steps. We substituted these line segments by a Brownian bridge using 6 endpoints (*i.e.*, 10 min. intervals) for each set of consecutive point locations $\boldsymbol{u}$. Let $\tau_{t0} = t$ and $\tau_{t6} = t + 1$. Then a Brownian bridge starting at x_t at time τ_{t0} and passing through point y_t at time τ_{t6}, $\tau_{t6} > \tau_{t0}$, is given by:

$$W_{\tau_{t0},x_t}^{\tau_{t6},y_t}(\tau_t) = \boldsymbol{x}_t + W(\tau_t - \tau_{t0}) - \frac{\tau_t - \tau_{t0}}{\tau_{t6} - \tau_{t0}} \cdot (W(\tau_{t6} - \tau_{t0}) - \boldsymbol{y}_t + \boldsymbol{x}_t)$$

And hence the resulting Wiener process at hour t can be written as the vector

$$W_{\tau_{t0},x_t}^{\tau_{t6},y_t}(\tau_t) = [\boldsymbol{u}_{g\tau_{t0}}, \boldsymbol{u}_{g\tau_{t1}}, \dots, \boldsymbol{u}_{g\tau_{t6}}]$$

2 Prior specification of hierarchical abundance models

For all the hierarchical abundance models (HMs) considered in our simulation study, we chose vaguely informative priors for detection parameters θ_{det}, *i.e.,* $Uniform(0,1)$, and a weakly informative $Cauchy(0,10)$ prior for the site-abundance λ. The Stan codes for each HM fitted in our study can be found online at https://github.com/MartijnUH/RWsim_abundance_models. These models were fitted using four parallel MCMC chains, with 2000 iterations, which included 1000 iterations that were discarded as burn-in iterations. In total our simulation study consisted of 900 different scenarios, for which we fit 40 models each (*i.e.,* one for each simulation replicate). To make it computationally efficient to fit all these models, we used the high-performance computing (HPC) clusters provided by the VSC (Flemish Supercomputer Center). We ran our models in parallel using 8 threads, with 36 nodes

per thread. Moreover, we broke up the likelihood statement in partial sums and divided the calculations over 8 threads per chain using Stan's *reduce_sum* built-in function.

3 Predictive performance, goodness-of-fit and MCMC convergence

To evaluate the predictive performance and the goodness-of-fit of competing HMs, we calculated respectively the leave-one-out expected log predictive density[1] (LOO ELPD; **Supplementary fig. S1**), and Bayesian *P*-values[2,3] (**Supplementary fig. S2**) in the generated quantities block of Stan. We derived the Bayesian *P*-values as follows: for each posterior sample, $s = 1, 2, ..., 4000$, we generated data $y_{ij}^{(s)}$ and retrieved expected values $E_{ij}^{(s)}$, then calculated

$$T^{(s)} = \sum_i \sum_j \frac{(y_{ij}^{(s)} - E_{ij}^{(s)})^2}{E_{ij}^{(s)}}$$

The proportion of samples for which $T^{(s)}$ exceeds the value of the same statistic, computed with actual data y_{ij} rather than $y_{ij}^{(s)}$, is the Bayesian *P*-value P_B. Note that expected values $E_{ij}^{(s)}$, needed to calculate $T^{(s)}$, depend on the HM,

$$E_{ij}^{(s)} = \begin{cases} 1 - \left(1 - p^{(s)}\right)^{N_i^{(s)}} & if \quad Bernoulli - Poisson\, mixture \\ N_i^{(s)} \cdot p^{(s)} & if \quad Binomial - Poisson\, mixture \\ N_i^{(s)} \cdot p^{(s)} & if \quad Poisson - Poisson\, mixture \end{cases}$$

Finally, we assessed the model convergence by the proportion of divergent chains (**Supplementary fig. S3**) and the $\hat{R}$-statistic (**Supplementary fig. S4**), and the sampling efficiency in the bulk (**Supplementary fig. S5**) and tail (**Supplementary fig. S6**) of the posterior distribution by the effective sample size

4 95% credible interval coverages and root mean square errors for model parameters

In the main paper, we show the direction of bias and the proportion of simulations yielding a |relative bias| < 0.5 for estimated detection parameters θ_{det}, abundances λ, site use frequencies λ_{use}, and trends in λ for all of the models. Here, we also show summary tables that display the 95% credible

interval (CI) coverages and root mean square errors for BernP, BP and PP models (**Supplementary tables S1, S2 and S3**).

5 Supplemental tables

For the additional scenario analyses where spatial variation in θ_{det} was induced according to a Matérn process, we only display the direction of the bias graphically in the main paper. Here we also show the proportion of simulations yielding a |relative bias| < 0.5 for estimated detection parameters θ_{det}, abundances λ, site use frequencies λ_{use}, and trends in λ for all of the models and covariate structures (**Supplementary tables S4 and S5)**.

6 Supplemental tables

Supplementary table S1. Summary table for estimator quality of detection parameters θ_{det} obtained from three Bayesian hierarchical models (BernP, BP, PP). Cells display the 95% CI coverage and the root mean square error based on forty simulation replicates (note that the highest CI coverages and lowest root mean square errors in each scenario are indicated in bold).

			**95% CI coverage**			**Root mean square error**		
**HRA (km^2)**	**N**	**ρ**	**BernP**	**BP**	**PP**	**BernP**	**BP**	**PP**
0.65 (closure)	600	0.7	0.00	2.50	0.00	0.16	0.04	0.27
		0.95	0.00	7.50	0.00	0.10	0.03	0.24
	300	0.7	0.00	2.50	0.00	0.16	0.05	0.27
		0.95	0.00	12.50	0.00	0.10	0.03	0.24
0.65	600	0.7	0.00	5.00	0.00	0.08	0.04	0.17
		0.95	0.00	27.50	0.00	0.07	0.03	0.18
	300	0.7	0.00	17.50	0.00	0.09	0.04	0.19
		0.95	0.00	32.50	0.00	0.07	0.03	0.20
2.61	300	0.7	0.00	32.50	0.00	0.05	0.02	0.11
		0.95	0.00	40.00	0.00	0.05	0.02	0.13
	150	0.7	0.00	35.00	0.00	0.05	0.03	0.13
		0.95	0.00	47.50	2.50	0.05	0.02	0.15
10.38	150	0.7	0.00	60.00	0.00	0.04	0.01	0.09
		0.95	0.00	77.50	0.00	0.04	0.01	0.09
	50	0.7	0.00	67.50	0.00	0.05	0.02	0.11
		0.95	0.00	85.00	7.50	0.05	0.01	0.10
41.58	30	0.7	0.00	70.00	0.00	0.04	0.01	0.08

	0.95	0.00	57.50	0.00	0.04	0.01	0.09
10	0.7	0.00	85.00	0.00	0.04	0.01	0.09
	0.95	0.00	77.50	2.50	0.05	0.01	0.12
**Median**		0.00	37.50	0.00	0.05	0.02	0.13

Supplementary table S2. Summary table for estimator quality of abundances λ obtained from three Bayesian hierarchical models (BernP, BP, PP). Cells display the 95% CI coverage and the root mean square error based on forty simulation replicates.

			95% CI coverage			Root mean square error		
**HRA (km^2)**	**N**	**ρ**	**BernP**	**BP**	**PP**	**BernP**	**BP**	**PP**
0.65 (closure)	480	0.7	0.00	0.00	5.00	1.83	10.22	1.30
		0.95	0.00	0.00	2.50	1.90	15.40	1.43
	240	0.7	0.00	0.00	7.50	0.98	4.27	0.69
		0.95	2.50	0.00	7.50	1.01	7.98	0.74
0.65	480	0.7	0.00	40.00	0.00	2.73	3.19	2.59
		0.95	0.00	30.00	0.00	2.86	8.91	2.76
	240	0.7	0.00	47.50	0.00	1.39	2.41	1.34
		0.95	0.00	37.50	0.00	1.43	8.50	1.40
2.61	240	0.7	5.00	7.50	7.50	0.90	6.09	0.83
		0.95	0.00	12.50	0.00	1.15	13.11	1.10
	120	0.7	2.50	20.00	12.50	0.48	3.63	0.44
		0.95	0.00	25.00	5.00	0.58	7.88	0.72
10.38	120	0.7	100.00	0.00	92.50	0.20	15.01	0.27
		0.95	20.00	2.50	45.00	0.39	11.26	0.39
	60	0.7	92.50	2.50	85.00	0.12	9.62	0.15
		0.95	32.50	22.50	65.00	0.22	6.79	0.61
41.58	60	0.7	17.50	0.00	17.50	0.76	17.48	0.74
		0.95	90.00	2.50	85.00	0.14	12.91	0.63
	30	0.7	37.50	0.00	32.50	0.32	11.97	0.50
		0.95	82.50	7.50	82.50	0.12	8.64	0.64
**Median**			2.50	5.00	7.50	0.83	8.78	0.73

Supplementary table S3. Summary table for estimator quality of site use frequencies λ_{use} obtained from three Bayesian hierarchical models (BernP, BP, PP). Cells display the 95% CI coverage and the root mean square error based on forty simulation replicates.

			95% CI coverage			Root mean square error		
HRA (km^2)	N	ρ	BernP	BP	PP	BernP	BP	PP
0.65 (closure)	480	0.7	0.00	0.00	5.00	1.83	10.22	1.30
		0.95	0.00	0.00	2.50	1.90	15.40	1.43
	240	0.7	0.00	0.00	7.50	0.98	4.27	0.69
		0.95	2.50	0.00	7.50	1.01	7.98	0.74
0.65	480	0.7	0.00	15.00	0.00	11.22	8.00	11.09
		0.95	0.00	30.00	0.00	11.35	8.05	11.26
	240	0.7	0.00	25.00	0.00	5.65	3.90	5.59
		0.95	0.00	42.50	0.00	5.69	7.31	5.65
2.61	240	0.7	0.00	42.50	0.00	11.54	6.69	11.46
		0.95	0.00	55.00	0.00	11.80	9.78	11.74
	120	0.7	0.00	37.50	0.00	5.83	3.79	5.78
		0.95	0.00	55.00	2.50	5.93	5.69	5.84
10.38	120	0.7	0.00	60.00	0.00	14.25	9.60	14.16
		0.95	0.00	80.00	0.00	14.70	6.92	14.58
	60	0.7	0.00	67.50	0.00	7.22	6.04	7.17
		0.95	0.00	87.50	5.00	7.41	3.64	7.21
41.58	60	0.7	0.00	72.50	0.00	18.77	10.11	18.73
		0.95	0.00	62.50	0.00	19.37	10.99	19.18
	30	0.7	0.00	80.00	0.00	9.38	6.40	9.29
		0.95	0.00	82.50	2.50	9.61	5.04	9.44
**Median**			0.00	48.75	0.00	8.40	7.12	8.25

Supplementary table S4. Summary table for estimator quality of detection parameters θ_{det}, abundances λ and site use frequencies λ_{use} obtained from three Bayesian hierarchical models (BernP, BP, PP). Cells display the proportion of simulation replicates that satisfy |Relative Bias| ≤ 0.5 for scenarios with σ = 300 (HRA = 2.61 km^2).

			**|Relative Bias| ≤ 0.5**								
			**Detection parameters θ_{det}**			**Abundance λ**			**Site use frequency λ_{use}**		
**θ_{det}**	**N**	**ρ**	**BernP**	**BP**	**PP**	**BernP**	**BP**	**PP**	**BernP**	**BP**	**PP**
constant	240	0.70	0.00	0.08	0.00	0.38	0.08	0.58	0.00	0.48	0.00
		0.95	0.00	0.20	0.00	0.05	0.08	0.08	0.00	0.45	0.00
	120	0.70	0.00	0.05	0.00	0.38	0.15	0.48	0.00	0.43	0.00
		0.95	0.00	0.20	0.00	0.10	0.13	0.13	0.00	0.30	0.03
**Median**			0.00	0.14	0.00	0.14	0.00	0.24	0.10	0.30	0.00
Spatial variation (naïve model)	240	0.70	0.03	0.68	0.00	0.58	0.00	0.78	0.03	0.50	0.00
		0.95	0.00	0.68	0.00	0.15	0.00	0.25	0.00	0.60	0.00
	120	0.70	0.03	0.55	0.00	0.75	0.00	0.75	0.00	0.30	0.03
		0.95	0.00	0.58	0.00	0.25	0.00	0.38	0.00	0.30	0.03
**Median**			0.01	0.63	0.00	0.41	0.00	0.56	0.00	0.40	0.01
Spatial variation (covariate model)	240	0.70	0.03	0.48	0.00	0.50	0.00	0.80	0.08	0.10	0.00
		0.95	0.00	0.70	0.00	0.18	0.00	0.43	0.00	0.23	0.00
	120	0.70	0.03	0.28	0.00	0.80	0.00	0.65	0.00	0.03	0.08
		0.95	0.00	0.45	0.03	0.28	0.00	0.43	0.00	0.03	0.10
**Median**			0.01	0.46	0.00	0.39	0.00	0.54	0.00	0.06	0.04

Supplementary table S5. Summary table for estimator quality of relative abundance (10% trend: $\lambda_N/\lambda_{0.9N}$ and 20% trend: $\lambda_N/\lambda_{0.8N}$) obtained from three Bayesian hierarchical models (BernP, BP, PP). Cells display the proportion of simulation replicates that satisfy |Relative Bias| ≤ 0.5 for scenarios with σ = 300 (HRA = 2.61 km^2).

			**|Relative Bias| ≤ 0.5**					
			**10% trend**			**20% trend**		
$\boldsymbol{\theta_{det}}$	**N**	$\boldsymbol{\rho}$	**BernP**	**BP**	**PP**	**BernP**	**BP**	**PP**
constant	240	0.70	0.53	0.49	0.53	0.73	0.73	0.72
		0.95	0.62	0.36	0.60	0.78	0.58	0.70
	120	0.70	0.58	0.56	0.52	0.71	0.53	0.66
		0.95	0.37	0.36	0.42	0.68	0.42	0.64
**Median**			0.55	0.43	0.53	0.72	0.56	0.68
Spatial variation (naïve model)	240	0.70	0.27	0.22	0.28	0.53	0.24	0.44
		0.95	0.28	0.18	0.29	0.58	0.38	0.48
	120	0.70	0.33	0.18	0.16	0.56	0.36	0.52
		0.95	0.36	0.21	0.25	0.43	0.34	0.29
**Median**			0.30	0.20	0.27	0.54	0.35	0.46
Spatial variation (covariate model)	240	0.70	0.23	0.18	0.26	0.48	0.41	0.38
		0.95	0.35	0.17	0.21	0.59	0.30	0.44
	120	0.70	0.28	0.18	0.23	0.59	0.38	0.53
		0.95	0.35	0.20	0.21	0.44	0.32	0.34
**Median**			0.32	0.18	0.22	0.54	0.35	0.41

7 Supplemental figures

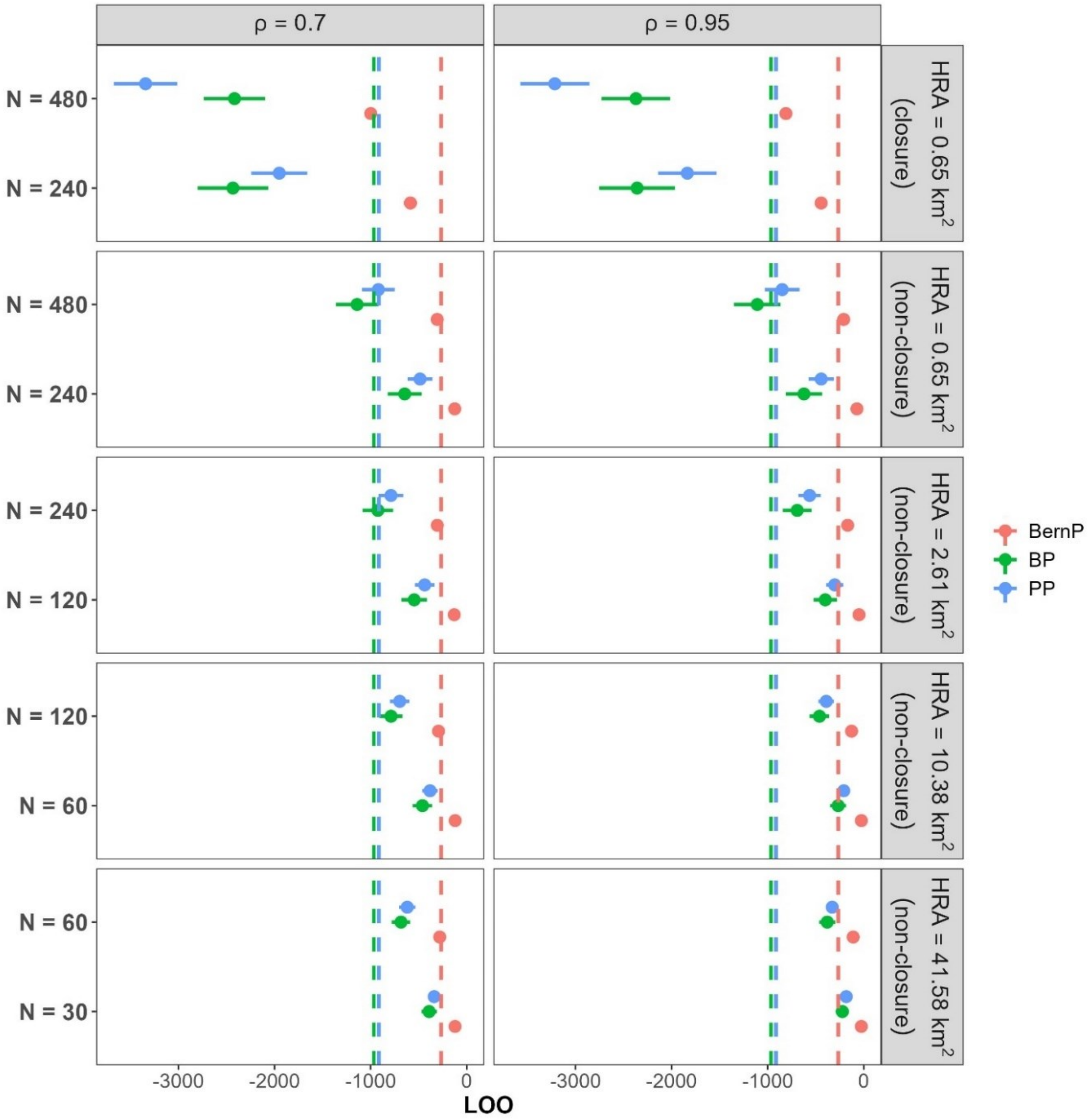

Supplementary figure S1. LOO for all combinations of model used (BernP, BP, PP), population size N (30, 60, 120, 240, 480), closure (closure, non-closure), movement parameters σ (150, 300, 600, 1200) and ρ (0.7, 0.95), and the emerging home range area in km^2 (0.65, 2.61, 10.38, 41.58). Dots and horizontal lines represent means and standard errors for LOO in each scenario, while the vertical dashed line indicates the average LOO overall.

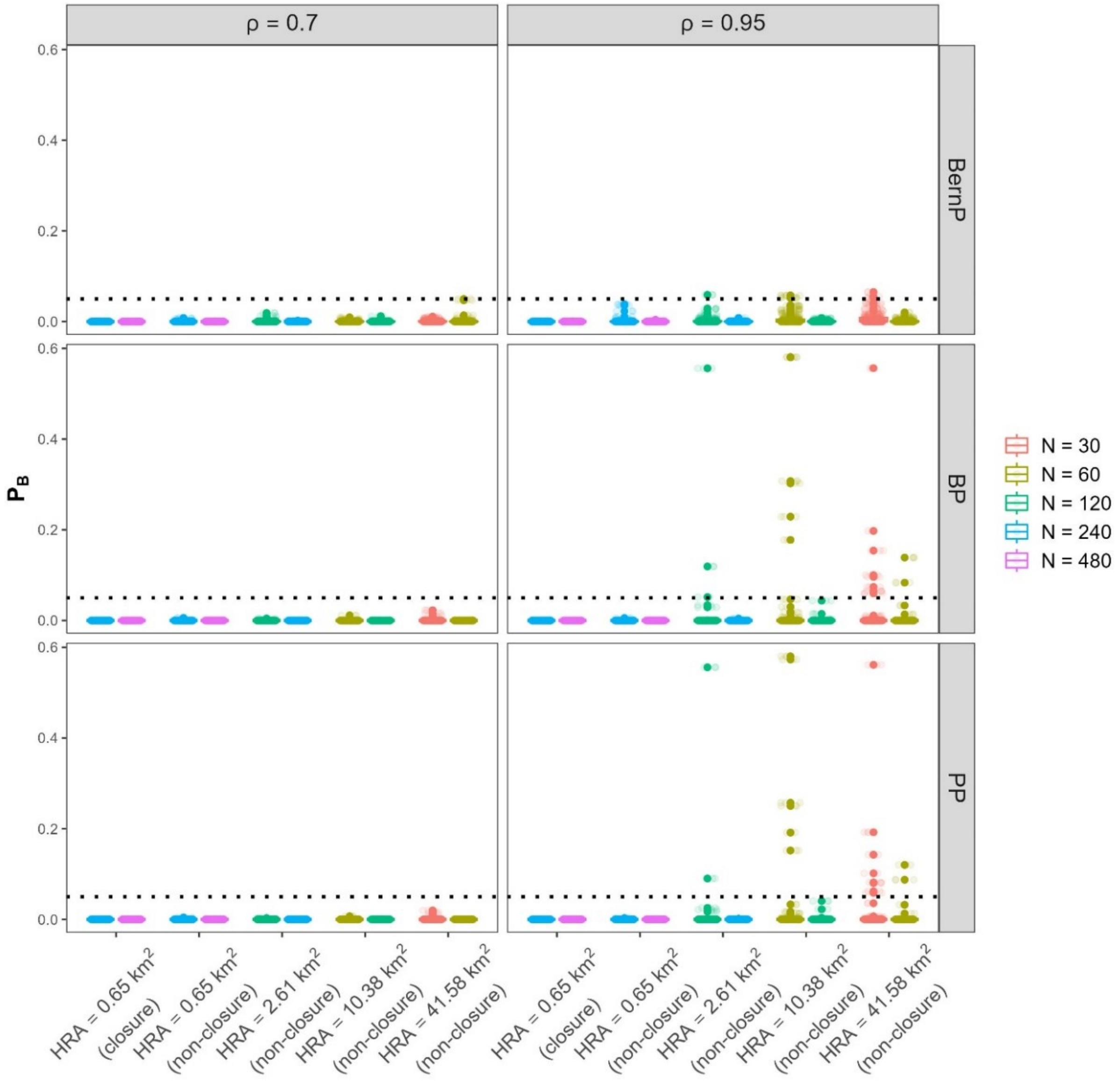

Supplementary figure S2. Bayesian P-values P_B for all combinations of model used (BernP, BP, PP), population size N (30, 60, 120, 240, 480), closure (closure, non-closure), movement parameters σ (150, 300, 600, 1200) and ρ (0.7, 0.95), and the emerging home range area in km^2 (0.65, 2.61, 10.38, 41.58.

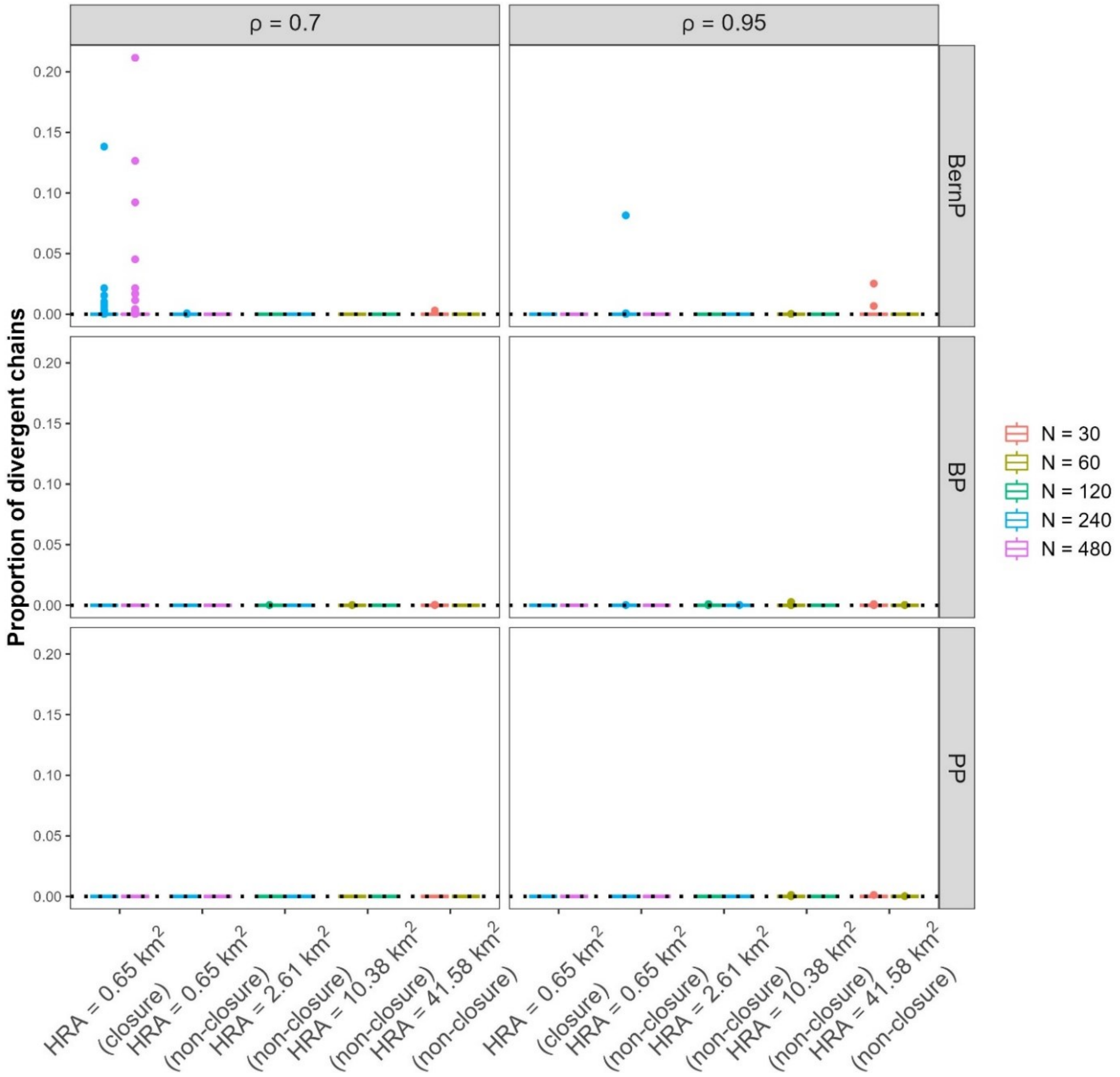

Supplementary figure S3. Proportion of MCMC chains that ended with a divergence for all combinations of model used (BernP, BP, PP), population size N (30, 60, 120, 240, 480), closure (closure, non-closure), movement parameters σ (150, 300, 600, 1200) and ρ (0.7, 0.95), and the emerging home range area in km^2 (0.65, 2.61, 10.38, 41.58).

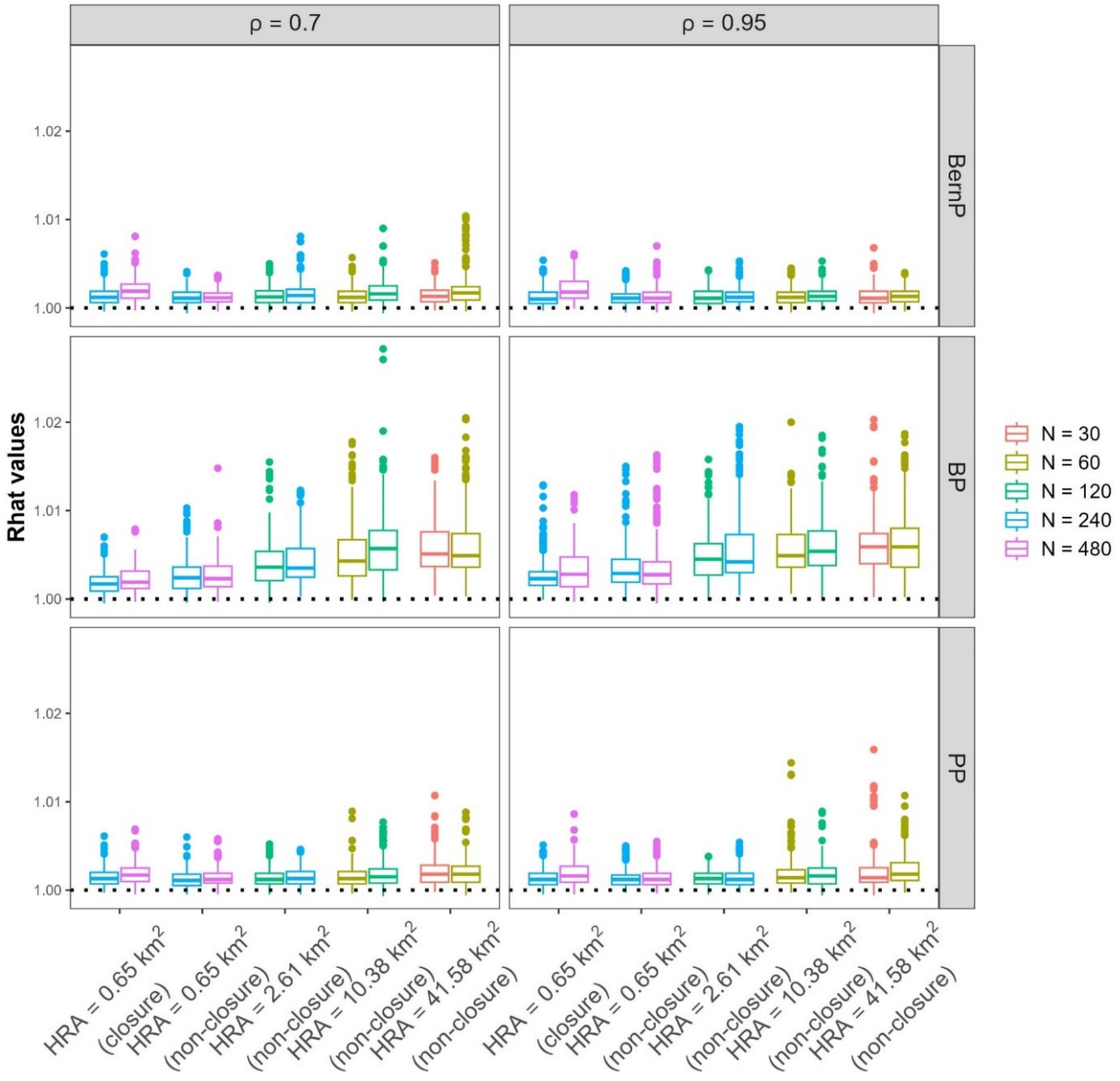

Supplementary figure S4. $\hat{R}$-statistic for all combinations of model used (BernP, BP, PP), population size N (30, 60, 120, 240, 480), closure (closure, non-closure), movement parameters σ (150, 300, 600, 1200) and ρ (0.7, 0.95), and the emerging home range area in km^2 (0.65, 2.61, 10.38, 41.58).

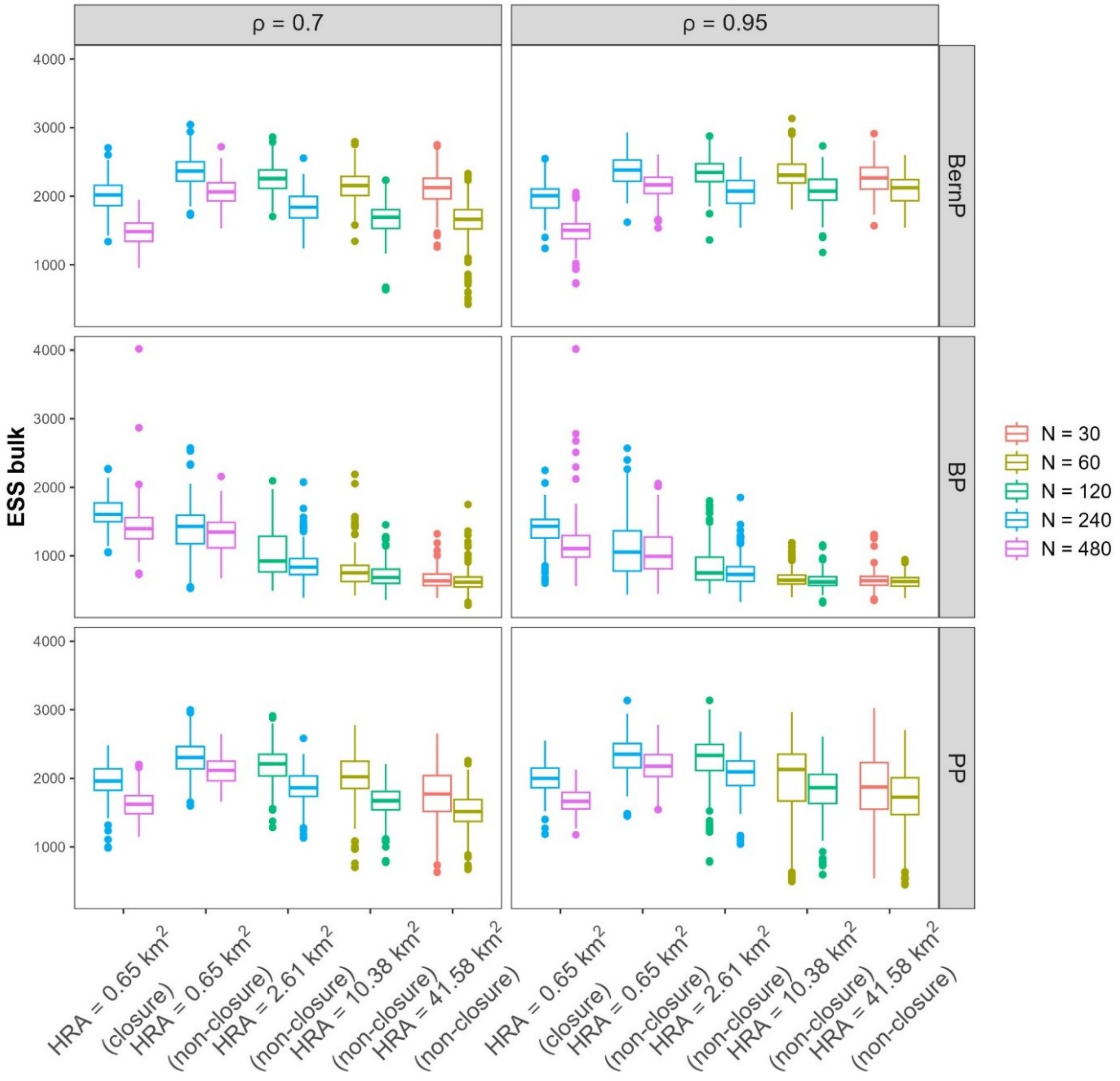

Supplementary figure S5. ESS bulk for all combinations of model used (BernP, BP, PP), population size N (30, 60, 120, 240, 480), closure (closure, non-closure), movement parameters σ (150, 300, 600, 1200) and ρ (0.7, 0.95), and the emerging home range area in km^2 (0.65, 2.61, 10.38, 41.58).

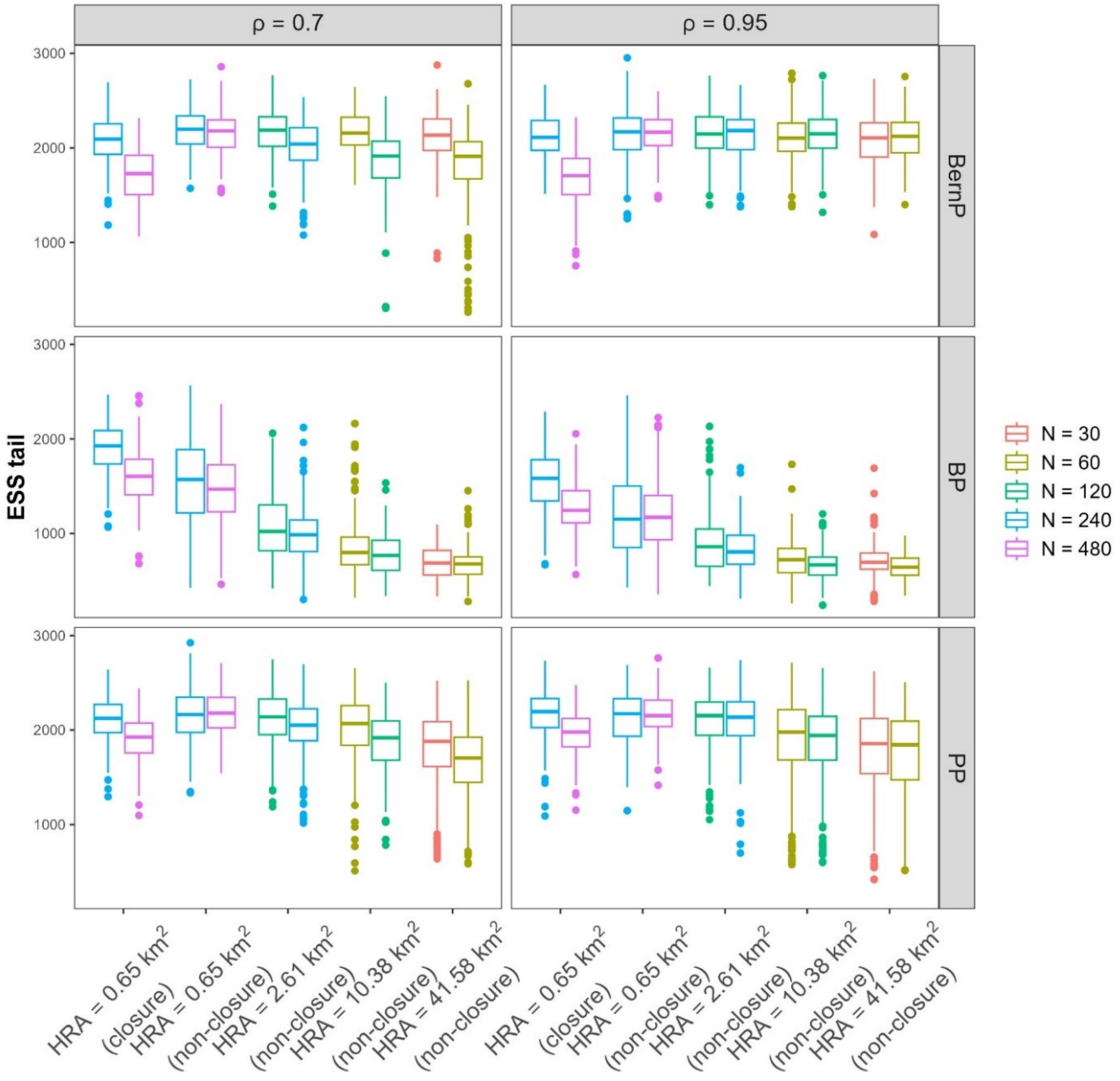

Supplementary figure S6. ESS tail for all combinations of model used (BernP, BP, PP), population size N (30, 60, 120, 240, 480), closure (closure, non-closure), movement parameters σ (150, 300, 600, 1200) and ρ (0.7, 0.95), and the emerging home range area in km^2 (0.65, 2.61, 10.38, 41.58).

References

1 Vehtari, A., Gelman, A. & Gabry, J. Practical Bayesian model evaluation using leave-one-out cross-validation and WAIC. *Statistics and Computing* **27**, 1413-1432. https://doi.org/10.1007/s11222-016-9696-4 (2017).

2 Link, W. A., Schofield, M. R., Barker, R. J. & Sauer, J. R. On the robustness of N-mixture models. *Ecology* **99**, 1547-1551. https://doi.org/10.1002/ecy.2362 (2018).

3 Hjort, N. L., Dahl, F. A. & Steinbakk, G. H. Post-Processing Posterior Predictive p Values. *Journal of the American Statistical Association* **101**, 1157-1174. https://doi.org/10.1198/016214505000001393 (2006).
